# Left lateral decubitus position during sedation‐free transnasal endoscopy: A pilot study

**DOI:** 10.1002/jpr3.70047

**Published:** 2025-06-11

**Authors:** Rose Lee, Yonna Oparaugo, Molly Mackensen, Katherine Vaidy

**Affiliations:** ^1^ Division of Pediatric Gastroenterology, Hepatology, and Nutrition, Department of Pediatrics Medical College of Wisconsin Milwaukee Wisconsin USA

**Keywords:** Dysphagia, Eosinophilic Esophagitis, Esophagoscopy, Pediatrics, Unsedated

## Abstract

**Objectives:**

Sedation‐free transnasal endoscopy (TNE) is a safe, feasible, and well tolerated procedure performed in children to evaluate the upper gastrointestinal tract. The procedural technique of TNE in children is adopted from procedural standards in adults, typically using the upright seated position. The left lateral decubitus (LLD) position may be preferred for optimal safety and visualization during TNE. This pilot study explored the feasibility and tolerance of TNE in pediatric patients using the LLD position.

**Methods:**

This was a retrospective review of 13 children who underwent sedation‐free TNE in the LLD position from October 2024 to February 2025 in an outpatient gastroenterology procedure suite. Procedure time, patient tolerance (TNEase score), adverse events, and patient demographics were collected and analyzed.

**Results:**

A total of 13 TNE procedures were successfully completed in the LLD position. The mean (standard deviation (SD)) age of the cohort was 12 years (2.7); 38% were female. The mean (SD) procedural time for esophagoscopy was 5.1 min (1.6). All patients had TNEase score of 2 or lower. Ten (77%) of patients had a TNEase score of 1. Two patients with history of anxiety and orthostasis experienced syncope in the upright seated position but subsequently completed the TNE in the LLD position without adverse events.

**Conclusions:**

LLD position for sedation‐free TNE is feasible and well tolerated in children. Findings should prompt further, prospective investigations of the benefits of LLD versus upright seated position, particularly in children with orthostatic intolerance.

## Abstract (highlights)

**What is Known:**

• Sedation‐free transnasal endoscopy (TNE) is a safe and well tolerated procedure for evaluating the upper gastrointestinal tract.
• Both the upright seated and left lateral decubitus position are standard positions for sedation‐free TNE in adults.
• There is no data on the safety and feasibility of left lateral decubitus position during TNE in children.

**What is New:**

• The left lateral decubitus position during sedation‐free TNE in children is feasible and well tolerated.
• This study indicates that left lateral decubitus may be the preferred position during TNE in children.

## 1 INTRODUCTION

Sedation‐free transnasal endoscopy (TNE) is a safe alternative method to sedated esophagogastroduodenoscopy (EGD) for evaluating the upper part of the gastrointestinal tract.^1^ Multiple studies have shown that TNE is minimally invasive, cost‐effective, and well tolerated.^1^, ^2^, ^3^ General anesthesia is uniformly used for sedated EGD in children. In 2016, the US Food and Drug Administration (FDA) issued a warning about potential adverse effects of repeated or prolonged exposure to general anesthetics on neurodevelopment of young children.^4^ For the past decade, there has been increasing interest in utilizing sedation‐free TNE for children with certain disorders such as eosinophilic esophagitis, which requires repeated endoscopic evaluations and recurrent exposure to the potential adverse effects of general anesthesia.^3^ TNE offers many advantages compared to sedated EGD such as decreased cost, convenience, short recovery time, safety and minimal invasiveness.^2^, ^5^, ^6^, ^7^ These factors translate to even larger indirect cost savings such as reduced work and school absenteeism.

Sedation‐free TNE is performed using a variety of ultra‐thin endoscopes with a sterile, single‐use, 3.5 mm outer diameter gastroscope. These endoscopes are FDA‐approved for use in children older than 4 years of age. TNE was first developed by adult gastroenterologists and many of their techniques were adopted by pediatric gastroenterologists.^8^ In adults, the TNE procedure is performed either in the upright seated or left lateral decubitus (LLD) position based on patient preference.^9^ In pediatrics, the upright seated position has been adopted without clear rationale while the LLD position has not been standardized.

There are no studies to date reporting on the feasibility and safety of the LLD position. The aim of this pilot study was to evaluate the feasibility and safety of the LLD position for the pediatric patients undergoing TNE.

**Figure 1:**
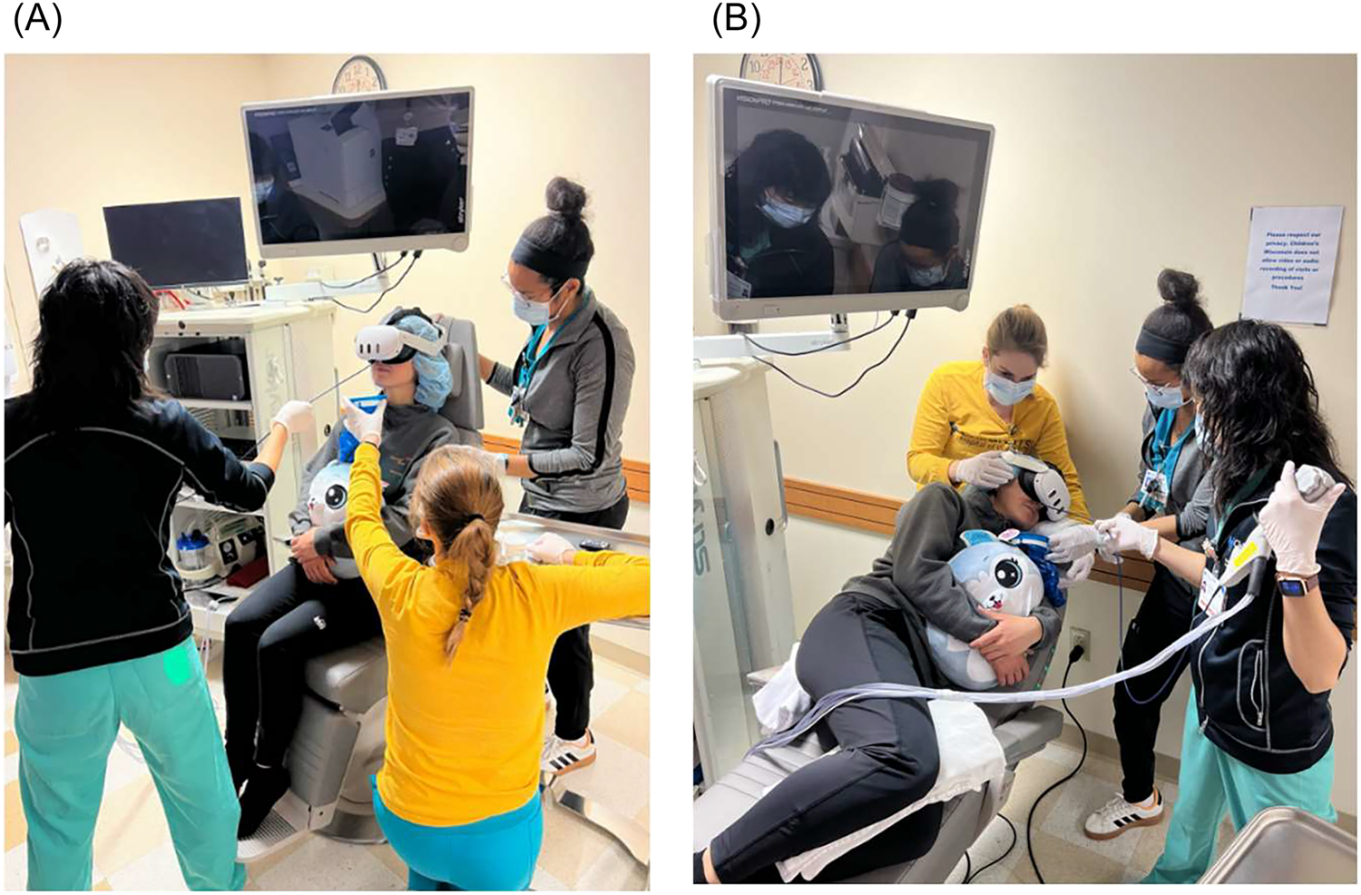
(A) Transnasal endoscopy (TNE) in the upright seated position. (B) TNE in the left lateral decubitus position.

## 2 METHODS

This was a retrospective review of all children who underwent sedation‐free TNE in the LLD position at Children's Wisconsin hospital, Milwaukee, WI, between October 2024 and February 2025 using data from the electronic medical records (EPIC). Transnasal endoscopies were performed by two experienced pediatric gastroenterologists, using a sterile, single‐use, 3.5 mm outer diameter, 85 cm or 110 cm long gastroscope with a 2 mm working channel. Patients were given the option to choose their preferred position for the TNE procedure, with both the upright and LLD positions offered to every individual (Figure 1). Everyone received the same preprocedural treatments including oxymetazoline to decrease nasal congestion and 4% lidocaine spray in the nose and back of the throat to reduce discomfort. Patients watched a video using virtual reality (VR) goggles during the entire procedure as a matter of distraction. Each TNE case was assessed with a “TNEase score” by the gastroenterologist after the completion of the TNE. The score was developed to evaluate patient tolerance and defined as: 1 = performed on patient with ease, 2 = performed with minimal complaints, 3 = performed with moderate complaints, 4 = performed but patient had significant complaints and resistance, 5 = patient did not tolerate, and procedure needed to be terminated.^10^ Data collection included TNE procedure time, adverse events, and TNEase score. Demographic information and indications for TNE were also collected for the study.

### 2.1 Ethics statement

The study was designed as a Quality Improvement (QI) project, and as such, it was exempt from Institutional Review Board (IRB) review.

## 3 RESULTS

Thirteen patients underwent the procedure in the LLD position (Table 1). Indications for TNE included eosinophilic esophagitis (*n* = 11), celiac disease (*n* = 3), and gastroesophageal reflux disease (*n* = 1). Two patients had both eosinophilic esophagitis and celiac disease. The mean age of the patients was 12 years, ranging from 7 to 16 years. Eleven TNE‐esophagoscopy (ES), one esophagogastroscopy (EG), and one EGD were performed based on the indications for TNE. Two patients who initially opted for the upright seated position had a syncopal episode during the procedure and chose to attempt the LLD position instead of terminating the procedure. TNE was subsequently completed in the LLD position without complications in these two patients.

**Table 1:** Patient characteristics and study findings.

Mean (SD) age (years)	12 (2.7)
Gender *n* (%)	
Male	8 (62%)
Female	5 (38%)
Race *n* (%)	
Caucasian	11 (84%)
Asian	1 (8%)
Hispanic	1 (8%)
Mean (SD) TNE procedure time (min)	
Esophagoscopy (*n* = 10)	5.1 (1.6)
Esophagogastroscopy (*n* = 1)	11.8
Procedure success rate *n* (%)	13 (100%)

Abbreviations: SD, standard deviation; TNE, transnasal endoscopy.

The mean TNE procedure time for ES was 5.1 min (*n* = 10) and EG was 11.8 min (*n* = 1). Equipment (scope and monitor) briefly malfunctioned during TNE‐EGD and one of the TNE‐ES cases. Therefore, accurate TNE procedure time for these two cases were not obtained. All 13 TNE cases, including the two cases with equipment malfunction, were completed successfully. Ten (77%) patients were graded as TNEase score of 1 (performed with ease) while three patients had a TNEase score of 2 (performed with minimal complaints). Of the three patients who received a TNEase score of 2, two experienced syncopal episodes, while the third case involved an equipment malfunction. Five of the thirteen patients previously had TNE in the upright seated position. All five of these patients expressed preference for the LLD compared to the upright seated position.

## 4 DISCUSSION

This is the first study exploring the feasibility and safety of the LLD position in the pediatric population for sedation‐free TNE. The upright seated and LLD position are the current standard of care procedural positions for sedation‐free TNE in adults.^9^ There is no prior data to provide support for a specific, preferred position in children. In this study, we demonstrated that the LLD position is safe and well tolerated for sedation‐free TNE in children.

All 13 cases performed in the LLD position were successfully completed. Other than the brief malfunctioning of the equipment during two cases, no other adverse events occurred. None of the patients experienced any of the commonly reported adverse events of TNE such as epistaxis, throat pain, or vomiting. Notably, two patients experienced syncope while seated upright during the procedure but successfully completed the procedure without any complaints or adverse events after being repositioned to the LLD position. Both patients had a previous history of syncope, dizziness, and anxiety. These observations may suggest that the LLD position could be superior to the upright seated position, particularly in patients with a history of orthostatic intolerance. Further, the LLD position was well tolerated in young children. The youngest patient in the study was only 7 years old and had a TNEase score of 1 (performed with ease). The chair used for TNE is an adjustable chair with adjustable support for arms and back. As it is designed for adults, the upright seated position may not be ideal for small children. The LLD position may be both safer and more comfortable for young children.

The average TNE procedure time for ES (5.1 min) was shorter than EG (11.8 min) as expected. We were unable to obtain the procedure time for TNE‐EGD due to mechanical malfunction. There are additional reasons for performing TNE in LLD position. When patients are placed in the LLD position, gastric secretions are collected in the gastric fundus, away from the pylorus, allowing for improved visualization of the pylorus compared to the seated upright position. Prompt identification of the pylorus is critical during TNE‐EGD to minimize patients' discomfort.

The main limitation of our study is its small sample size from a single center and retrospective study design. The strength of this study includes examining all cases performed in the LLD position over the study period to minimize selection bias. Future studies should prospectively investigate a larger sample size to further validate the feasibility, safety and procedural time of the LLD position compared to the upright seated position.

## 5 CONCLUSION

In summary, the LLD position during sedation‐free TNE in children is feasible, safe, well tolerated, and potentially superior to the upright seated position, particularly in children with orthostatic intolerance.

## CONFLICT OF INTEREST STATEMENT

Rose Lee and Katherine Vaidy serve as consultants for EvoEndo Inc.

## References

[1] Dumortier J , Napoleon B , Hedelius F , et al. Unsedated transnasal EGD in daily practice: results with 1100 consecutive patients. Gastrointest Endosc. 2003;57:198‐204. 10.1067/mge.2003.59 12556784

[2] Nguyen N , Lavery WJ , Capocelli KE , et al. Transnasal endoscopy in unsedated children with eosinophilic esophagitis using virtual reality video goggles. Clin Gastroenterol Hepatol. 2019;17:2455‐2462. 10.1016/j.cgh.2019.01.023 30708107 PMC6663663

[3] Sabe RMM , Elzayat A , Buckley A , Shah JR , Khalili AS , Sferra TJ . Transnasal endoscopy for children and adolescents with eosinophilic esophagitis: a single‐center experience. Gastroenterol Res. 2022;15:155‐161. 10.14740/gr1535 PMC9451578 36128188

[4] Andropoulos DB , Greene MF . Anesthesia and developing brains ‐ implications of the FDA warning. N Engl J Med. 2017;376:905‐907. 10.1056/NEJMp1700196 28177852

[5] Venkatesh RD , Leinwand K , Nguyen N . Pediatric unsedated transnasal endoscopy. Gastrointest Endosc Clin N Am. 2023;33:309‐321. 10.1016/j.giec.2022.10.006 36948748

[6] Thakkar K , El‐Serag HB , Mattek N , Gilger MA . Complications of pediatric EGD: a 4‐year experience in PEDS‐CORI. Gastrointest Endosc. 2007;65:213‐221. 10.1016/j.gie.2006.03.015 17258979

[7] Lightdale JR , Liu QY , Sahn B , Troendle DM , Thomson M , Fishman DS . Pediatric endoscopy and high‐risk patients: a clinical report from the NASPGHAN endoscopy committee. J Pediatr Gastroenterol Nutr. 2019;68:595‐606. 10.1097/MPG.0000000000002277 30664560 PMC8597353

[8] Leisser A , Delpre G , Kadish U . Through the nose with the gastroscope. Gastrointest Endosc. 1990;36:77. 10.1016/s0016-5107(90)70939-x 2311893

[9] Parker C , Alexandridis E , Plevris J , O'Hara J , Panter S . Transnasal endoscopy: no gagging no panic! Frontline Gastroenterol. 2016;7:246‐256. 10.1136/flgastro-2015-100589 28839865 PMC5369487

[10] Nguyen N , Pan Z , Smith C , Friedlander JA . Transnasal endoscopy ease score “TNEase score” to evaluate patient tolerance of unsedated transnasal endoscopy. J Pediatr Gastroenterol Nutr. 2024;78:381‐385. 10.1002/jpn3.12102 38374574

